# The Future of the Hospitals

**Published:** 1920-11-27

**Authors:** Ernest Rumbelow

**Affiliations:** Organising Secretary to the Weekly Subscribers' Fund, Kent Ophthalmic Hospital, Maidstone


*To the Editor of* The Hospital.

### "The Future of the Hospitals."

*To the Editor of* The Hospital.

Sir,—After reading various appeals in the daily papers for the hospitals throughout our country, more especially in London and the provinces, no doubt all persons interested in this grand work are wondering what the future of our voluntary institutions will be, so may I state what (with the hearty co-operation of the employers of labour) the workers have done for the Kent County Ophthalmic Hospital at Maidstone?

A scheme was organised in 1918, called the "Weekly Subscribers' Fund," to which the employees of various firms paid weekly contributions, in most cases deducted from wages—by signing a slip of paper the workers gave the employers authority to make the deduction. In this way we hope to raise £5,000 this year, compared with £200 in 1918.

At the last annual meeting of the County Hospital the President, Lord Harris, said:

"With such a scheme in vogue as obtained at that institution he could not see why the hospitals of England should be under any apprehension as to the future. What they were doing in Kent every hospital could do. He was proud to be connected with an institution which had done such practical good work, and one which distributed such an enormous amount of skilled treatment to those who suffered the most trying form of illness possible—the deprivation of sight and hearing."

Now, Sir, much more would be done if employers of labour would agree to deduct the amount from wages when it is the wish of the workers. My own experience in organising is that the men prefer to have the amount deducted from wages. "What they never have they never miss," and where the amount is not deducted from wages, my greatest trouble is finding a voluntary collector.

So may I appeal to all employers of labour throughout the country to kindly help in this noble cause of supporting these grand institutions in this way? It only requires a lead, and our voluntary system of supporting hospitals will be saved.

One firm in Kent gave about £39 per annum to their hospital; now it is deducted from wages it will realise £592 per annum. Another firm, £396, compared with £20. We have over 27,000 members now paying weekly, and in return they receive benefits for themselves and their dependants. They also take part in the management of the County Hospital, and they work together in harmony one with another, or what one might call "a happy family." A good number of these subscribers are railway men, who contribute 3d. per week, which entitles them to the benefits at the hospitals, Surgical Aid Society, and Railway Convalescent Home, and thousands of others would like to have this advantage if only it could be deducted from wages.

The railwaymen will be subscribing this year over £1,000, compared with £170 last year, and Lloyd's Paper Mills, Sittingbourne, will contribute nearly £700 in the next twelve months, to be divided between St. Bartholomew's Hospital, Rochester, and the Maidstone Hospital.

The workers are certainly right in disdaining charity, and insisting on paying for themselves and in their own way. It is not merely an insurance in helping themselves —they are helping others who are not organised, and need the help of those who are able to give. It is, however, quite clear that a purely charitable system could not cope with modern requirements, nor does it satisfy the mind of the great body of workers to-day. If there were no other alternative between pure charity and State maintenance, there is no doubt that the future must lie in the latter direction. But the scheme adopted by the Kent County Ophthalmic Hospital these last two years clearly shows that we do *not* want State control.

So let us keep clear of the State hospital, and hold fast to the voluntary principle. There is always a better atmosphere in a voluntary system, but it must be efficient, and I trust the Minister of Health, Dr. Addison, will take note of what the workers are doing, and what they are willing to do, if only we could have the *unanimous* and hearty co-operation of the employers of labour.

Yours faithfully,

ERNEST RUMBELOW,

Organising Secretary to the Weekly Subscribers' Fund, Kent Ophthalmic Hospital, Maidstone.

November 22, 1920.

